# SPME-based investigation of thapsigargin-induced alterations in the volatilome of human melanoma cells

**DOI:** 10.1007/s00216-026-06359-8

**Published:** 2026-02-04

**Authors:** Elisabetta Santarelli, Matteo Delli Carri, Maria Rosaria Miranda, Vicky Caponigro, Vincenzo Vestuto, Agnieszka Smolinska, Andrea Manni, Pietro Campiglia, Giacomo Pepe, Carlo Crescenzi

**Affiliations:** 1 Department of Pharmacy, University of Salerno, 84084 Fisciano, Salerno, Italy; 2 PhD Program in Drug Discovery and Development, University of Salerno, 84084 Fisciano, Salerno, Italy; 3 Department of Pharmacology and Toxicology, Maastricht University, Maastricht, The Netherlands; 4 Spectra 2000 Srl, 00133 Rome, Italy

**Keywords:** Cell volatilome, Solid-phase microextraction, Headspace, Volatile organic compounds, Chemometrics, Classification

## Abstract

This study explores the feasibility of using headspace solid-phase microextraction (HS-SPME) combined with gas chromatography-mass spectrometry (GC-MS) to analyse the volatilome of human melanoma A375 cells. The goal was to identify and characterise the volatile organic compounds (VOCs) and monitor alterations induced by treatment with thapsigargin (TG), a drug known to disrupt calcium homeostasis, thereby inducing endoplasmic reticulum stress and ultimately triggering cell death. Reproducibility of experimental conditions is a major issue in biological experiments, which presents intrinsic variability of the samples to be analysed. In our case, initial analysis revealed a significant batch effect, accounting for 56.88% of the total variance. To address this, external parameter orthogonalisation (EPO) was applied, which successfully reduced the batch variance to just 0.16%. After this correction, the treatment factor became the dominant source of variation, explaining 47.12% of the total variance with strong statistical significance (*p*-value = 0.001). A supervised classification model using partial least squares discriminant analysis (PLS-DA) was developed and validated to characterise the differences between treated and untreated cells. The model achieved a mean overall accuracy of 92.21% and an area under the curve (AUC) of 0.974, indicating excellent discrimination between the two classes. The robustness of these findings was confirmed by repeated double cross-validation and permutation testing, which showed that the model’s predictive ability was not due to random chance. The results demonstrate that TG treatment induces a reproducible and highly discriminant volatilome signature in A375. This suggests that VOCs could potentially serve as biomarkers for monitoring cellular responses to drug treatments.

**Graphical abstract:**

Graphical abstract created with BioRender.com

**Figure:**
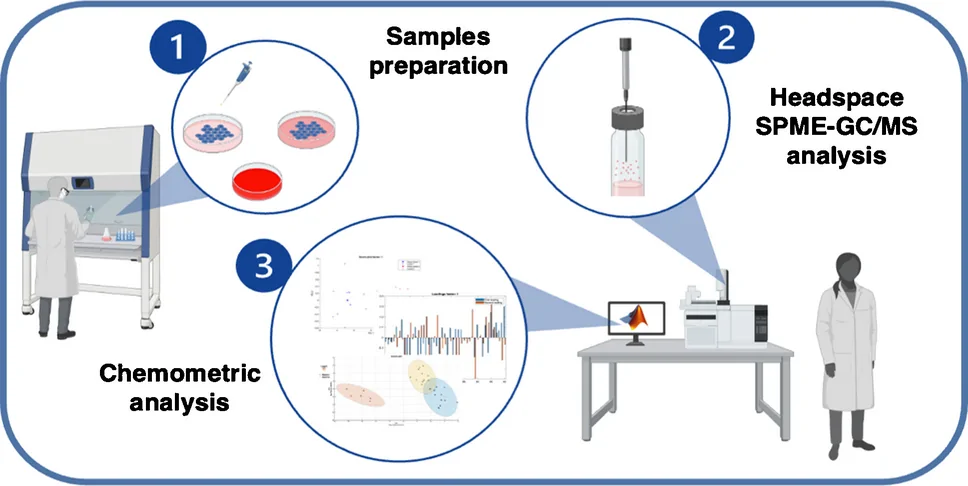

**Supplementary Information:**

The online version contains supplementary material available at https://doi.org/10.1007/s00216-026-06359-8.

## Introduction

The *volatilome* is the whole set of volatile compounds produced by a biological system, measurable through volatile organic compounds (VOCs). These are carbon-based compounds characterised by low molecular weights and low boiling points that arise as byproducts of cellular metabolism [1, 2]. Although exhaled breath is the primary route of VOCs’ elimination, they can also be detected in other biological matrices, including blood, urine, faeces, sweat, and tissues [3–7]. VOCs can therefore be sampled from the headspace (HS) of these specimens in vitro*,* and from the HS of cell cultures [8–11].

Because the VOC composition reflects the metabolic state of cells, alterations in metabolic pathways are often mirrored in the volatilome [12, 13]. In this context, cell cultures provide a controlled matrix in which biological variability (e.g., age, gender, diet, etc.) is minimised, enabling precise control over experimental conditions, and generating reproducible results at relatively low cost [14–16]. These advantages make them suitable for investigating drug responses, evaluating treatment efficacy, and identifying candidate metabolic biomarkers [7, 17, 18]. However, in vitro models face several methodological challenges that can influence the reliability and reproducibility of the results. These include the variability in the composition of the culture media and supplements, and differences in proliferation rates between cell cultures, influenced by growth conditions and passage number, that may influence metabolite concentration and ultimately lead to batch effect; other factors are the risk of introducing exogenous compounds during protocols and the practical difficulty of obtaining sufficient cell numbers for analysis [14–16]. Careful experimental design, including blank analysis, and standardised procedures are therefore essential to minimise these sources of variability. Batch effect correction in cellular headspace analysis is particularly challenging due to the absence of suitable internal standards [19] and the dynamic nature of VOC exchange between cells and the surrounding environment. These aspects highlight the importance of integrating statistical approaches with rigorous experimental controls.

Generally, tumour cells undergo extensive metabolic reprogramming, including changes in energy metabolism, biosynthetic pathways, and redox balance, often resulting in increased oxidative stress. Elevated levels of reactive oxygen species (ROS) can induce lipid peroxidation and oxidative modification of proteins and other cellular components, leading to the production of specific VOCs, such as alkanes, aldehydes, and alcohols. Elevated oxidative stress in cancer cells has also been associated with the overexpression and increased activity of the cytochrome P450 oxidase enzyme family [20–22]. Together, these processes can create a distinct VOC profile that reflects underlying metabolic and oxidative alterations, providing a rationale for using VOCs to study and potentially detect cancer-related metabolic changes.

A commonly used technique for VOCs’ collection and pre-concentration is solid-phase microextraction (SPME). In SPME, a fused-silica fibre, coated with a liquid, solid, or a combination of extraction phases, is exposed either directly to the sample (e.g., liquid) or to the gaseous headspace above it (HS-SPME). Volatile molecules partition into the coating depending on their polarity and molecular weight. The fibre is then introduced into the heated injector of a gas chromatograph, where analytes are thermally desorbed, separated, and detected, typically by gas chromatography-mass spectrometry (GC-MS). HS-SPME is solvent-free, requires minimal sample handling, and can be automated, thereby offering high reproducibility and sensitivity while preserving sample integrity.

This study evaluates the feasibility of using HS-SPME/GC-MS for characterising the VOC profile of human melanoma A375 cells and for monitoring alterations induced by pharmacological treatments. As a model drug, thapsigargin (TG) was selected. TG is a well-known and potent inhibitor of the sarco/endoplasmic reticulum Ca^2+^-ATPase (SERCA). By blocking this pump, TG increases cytoplasmic Ca^2+^, disrupting calcium homeostasis and subsequently triggers signalling cascades that promote different forms of cell death, including apoptosis [23, 24]. Unlike many anticancer agents that act on multiple and heterogeneous molecular targets, TG exerts a highly specific and well-defined mechanism of action, making it an ideal reference compound to investigate the cellular consequences of calcium dysregulation.

The human A375 melanoma cell line was chosen as an experimental model because it is a widely used and well-characterised representation of malignant melanoma, a highly aggressive cancer with limited therapeutic options and a strong need for improved diagnostic and monitoring strategies. A375 cells have been extensively studied in the context of calcium signalling, endoplasmic reticulum stress, and apoptotic pathways, making them particularly suitable for investigating the cellular effects of TG and allowing direct comparison with previously reported molecular and pharmacological data. While TG pharmacological activity and molecular mechanisms of action have been extensively studied in various types of cancer cells, including the A375 melanoma cell line [23, 25–31], its impact on the volatile profile of cells has not been investigated.

Volatile organic compounds released by cancer cells reflect metabolic and stress-related alterations and represent a promising source of non-invasive biomarkers for cancer detection and therapy monitoring. However, little is known about how drug-induced perturbations of calcium homeostasis and endoplasmic reticulum function influence the volatile profile of melanoma cells. This study addresses this gap by providing, to the best of our knowledge, the first comprehensive characterisation of the volatilome of A375 melanoma cells under basal conditions and following TG exposure. By linking TG-induced cellular stress and cell death to specific changes in volatile emissions, this work aims to improve the understanding of the metabolic consequences of calcium dysregulation and to identify potential volatile markers associated with drug response in melanoma cells.

## Materials and methods

### Cell culture and drug treatment

The human melanoma A375 cell line was obtained from the American Type Culture Collection (ATCC, Manassas, VA, USA). Cells were cultured in Dulbecco’s Modified Eagle Medium (DMEM) containing 4500 mg/mL glucose, supplemented with 10% (v/v) fetal bovine serum, 2 mM L-glutamine, 100 U/mL penicillin, and 0.1 mg/mL streptomycin. Cultures were maintained in Corning culture dishes (Corning, NY, USA) under standard conditions in a humidified incubator with 5% CO_2_ at 37 °C. Cells were routinely split every two days using a solution of 0.25% trypsin and 0.1% EDTA and then sub-cultured for further experiments. For each assay, cells were seeded in fresh complete medium and treated with thapsigargin (Sigma-Aldrich, St. Louis, MO, USA). Cell growth and viability were monitored using phase-contrast microscopy and trypan blue staining [32]. All experiments were performed in triplicate and repeated two times to ensure reproducibility and reliability of the results.

### Sample preparation

A375 cells were seeded in 100 mm Petri dishes at a density of 2 × 10^6^ cells per dish. After 24 h of seeding, cells were treated with TG at a final concentration of 600 nM for 6 h. The concentration and exposure time were selected based on preliminary experiments: cell viability was first assessed by MTT assay, as previously reported [33], after 24 h of TG exposure to determine the maximal concentration that did not cause excessive cytotoxicity (Fig. S1). Subsequently, Thioflavin T fluorescence assays were performed at the highest non-toxic concentration to evaluate the kinetics of ER stress induction (Fig. S2). The procedure was reported in our previous work [34]. From these analyses, 600 nM for 6 h was identified as the optimal condition to induce ER stress while maintaining acceptable cell viability. Control dishes received only the vehicle (0.1% DMSO) in the same volume used for the treated samples. After the 6 h treatment, cells were washed twice with phosphate-buffered saline (PBS), detached using trypsin, and resuspended in 5 mL of culture medium. Cells were then counted using a Bürker chamber. For each dish, 1 × 10^6^ cells were transferred into a 20 mL glass vial, and an additional 1 × 10^6^ cells from the same dish were placed into a separate glass vial, each adjusted to a final volume of 2 mL. The vials were tightly sealed with appropriate caps and incubated overnight (18 h) in a humidified incubator at 37 °C with 5% CO_2_, using the same conditions as for standard cell culture, prior to volatilome analysis. Blank culture mediums (2 mL, without cells) were incubated alongside the cells, under the same conditions.

A total of 12 control cell samples (CM), 12 TG-treated cell samples (TG), and 6 blank culture media (M) were prepared in two different batches, each consisting of 6 CM, 6 TG, and 3 M. The two batches were prepared independently over a week, following the same protocol.

### HS-SPME sampling

SPME sampling protocol applied was based on the method described by Beccaria et al. [35] with some modifications. Briefly, prior to extraction, samples were maintained at thermal equilibrium using a thermostatic agitator and constant magnetic stirring for 10 min at 37 °C.

For the extraction of volatile compounds, a SPME device equipped with a 10 mm fibre coated with 50/30 μm DVB/CAR/PDMS (divinylbenzene/carboxen/polydimethylsiloxane) from ThermoFisher Scientific (Waltham, MA, USA) was selected. The fibre was conditioned prior to use at 250 °C for 10 min. Following conditioning, the fibre was inserted into the vials and exposed to the headspace for 20 min at 37 °C to adsorb the volatile compounds. Finally, the SPME fibre was transferred to the GC injector port, which was maintained at 250 °C in splitless mode for the 5 min desorption phase.

### GC-MS analysis

GC-MS analysis was performed on an Agilent 8890 chromatograph system coupled to an Agilent 5977B MSD (Mass Selective Detector) from Agilent Technologies (Palo Alto, CA, USA). The separation of analytes was achieved on (5%-phenyl)-methyl-polysiloxane (HP-5MS UI, Agilent Technologies, Palo Alto, CA, USA) capillary column (40 m, 0.18 mm i.d., 0.18 μm f.t.). The flow rate of the helium carrier gas was maintained at 1 mL/min. The temperature programming of the GC oven began with an initial temperature of 40 °C (held for 5 min), followed by a linear increase to 150 °C at a rate of 6 °C/min, and then further increased linearly to 250 °C at a rate of 10 °C/min (held for 3 min). The total run time was 36 min, with a post-run temperature held at 280 °C for 1 min, and an additional 5 min of equilibration time before the next sample injection.

Electron ionisation (EI) was maintained at a constant energy of 70 eV, while the temperatures of the source and quadrupole mass analyser were set at 230 °C and 150 °C, respectively. MSD transfer line temperature was set to 280 °C. Full scan acquisition mode was employed, monitoring mass/charge (m/z) fragments between 35 and 350.

Each batch was analysed independently over one week, using the same operating conditions.

### Data pre-processing and statistical analysis

GC-MS data were pre-processed using the Mass Hunter ProFinder software (version B.08.00, Agilent Technologies, Palo Alto, CA, USA) to minimise the influence of systematic variation and noise [36]. Steps included chromatogram alignment, peak detection, deconvolution, and integration. Tentative identification of the volatile compounds was achieved by comparison of the experimental MS spectra and those provided by the spectral library database (NIST 2017, NIST MS Search 2.0), retaining peaks with a score of 80% and excluding features associated with column bleeding, empty glass vials, and fibre contamination [4, 5].

Statistical analyses were carried out using MATLAB 2025 (The MathWorks Inc., Natick, MA, USA), combining custom and built-in functions. Data were first log-10 transformed, and, before multivariate analysis, autoscaled (unit variance) [37, 38].

Exploratory unsupervised multivariate analysis was performed using principal component analysis (PCA) [39] on the autoscaled data. Score plots included 95% Hotelling’s *T*^2^ ellipses to assess sample distribution, group separation, and potential outliers. Loadings plots highlighted the most relevant variables, with significance assessed by parametric (*t*-test, ANOVA) and non-parametric (Mann-Whitney *U*, Kruskal-Wallis) tests, after normality evaluation with the Lilliefors test. *p*-values were corrected for multiple testing using the Benjamini-Hochberg (BH) procedure (FDR) [40].

To reduce batch-related variation, the first two components were removed with external parameter orthogonalisation (EPO), which separates biologically relevant information from systematic noise [41].

To evaluate the effectiveness of batch-related variability removal and to assess the impact of the TG treatment on the cells, ANOVA simultaneous component analysis (ASCA) was applied [42, 43]. ASCA combines analysis of variance (ANOVA) with PCA. First, ANOVA is used to partition the total variation in the dataset into contributions from each experimental factor, batch, and treatment in this study. Then, separate PCA models are applied to each component to explore underlying patterns and interpret the multivariate effects of each factor. Permutation tests (1000 randomisations) were performed to assess the significance of each factor and their interaction [44].

In order to evaluate group separation and identify the variables contributing to class discrimination, supervised classification was performed using partial least squares discriminant analysis (PLS-DA) [45] within a repeated double cross-validation (rDCV) framework (100 runs) [46]. Pre-processing steps (autoscaling and EPO) were embedded in each resampling loop to prevent information leakage. In each run, samples were randomly split into six outer folds; one fold was held out while the remaining were used for model training. Latent variables (up to 10) were selected by five-fold inner CV, minimising classification error. Models were refitted on full outer-training data, and probabilistic predictions were generated for the unseen fold. Outputs across runs yielded confusion matrices, probability scores, and performance statistics.

Model significance was tested by label permutation (1000 iterations) under the full validation design. Empirical *p*-values were derived as the fraction of permuted results exceeding observed metrics (*p* < 1/1000). Performance was reported as accuracy, sensitivity, specificity, and ROC-AUC, averaged across 100 runs. ROC curves were obtained from posterior probabilities; mean and 95% envelopes were computed from run-wise distributions. Variable importance was assessed by VIP scores (threshold = 1), with empirical 95% intervals across DCV fits. Stability of variable rankings was quantified by rank product analysis against permutation nulls. Directionality was inferred from averaged discriminant weights, retaining coefficients with interval estimates not crossing zero. Per-fit outputs (scores, loadings, coefficients, predictions) were stored, and uncertainty bands were obtained from empirical percentiles.

Finally, univariate analyses were applied to selected discriminant variables to compare trends across treatments and blank controls. *p*-values were calculated by the Mann-Whitney *U* test, corrected with the BH procedure. Significance was set at *p* < 0.05.

## Results and discussions

### Batch effect correction

**Fig. 1:**
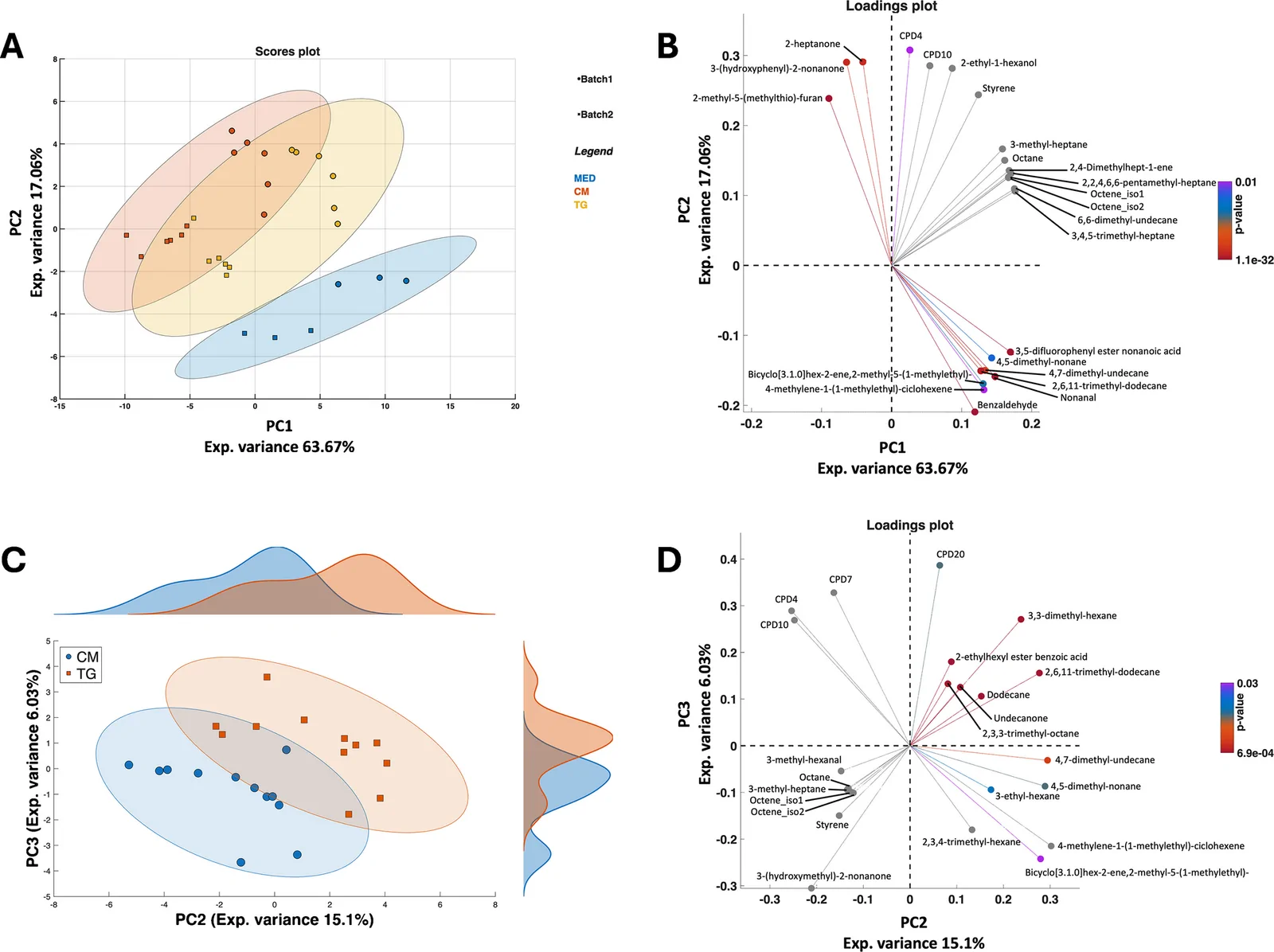
**A** PCA scores plot showing the distribution of data along PC1 and PC2, which explain respectively 63.67% and 17.06% of the total variance, before applying batch correction. Score plots legend: culture medium (MED) blue, non-treated cells (CM) red, treated cells (TG) yellow. Batch 1 circles, Batch 2 squares. The confidence ellipses are based on Hotelling’s *T*^2^ statistic for a 95% confidence interval. They show the variability within each group and illustrate the distribution of data along the primary axes, indicating the direction of maximum variance. **B** PCA loadings plot highlighting the variables most responsible for group separation in the multivariate space along PC1 and PC2, displaying those with higher contribution to variance, coloured according to their *p*-values. **C** PCA scores plot showing the distribution of CM and TG samples along PC2 and PC3, which explain respectively 15.1% and 6.03% of the total variance, before batch correction. Score plot legend: non-treated cells (CM) blue, treated cells (TG) red. The confidence ellipses are based on Hotelling’s *T*^2^ statistic for a 95% confidence interval. They show the variability within each group and illustrate the distribution of data along the primary axes, indicating the direction of maximum variance. **D** PCA loadings plot showing the contribution of each VOC to PC2 and PC3, displaying those with higher contribution to variance, coloured according to their *p*-values

A preliminary PCA revealed that PC1, explaining 63.67% of total variance, is primarily associated with differences between the two experimental batches (Fig. 1A). This batch effect is expected as samples were prepared and analysed in different weeks. Such non-biological variability, introduced at various stages of the experimental process (e.g., culture conditions, sample preparation, instrumental analysis), can lead to systematic differences that may mask the true biological effects under investigation [47]. Despite that, it is not always possible to fully minimise batch effects in laboratory practice.

The loadings plot (Fig. 1B) indicated that, among the variables most strongly correlated with PC1, 5 compounds, 3,5-difluorophenyl ester, nonanoic acid, 2,6,11-trimethyl-dodecane, 4,7-dimethyl-undecane, nonanal, and benzaldehyde showed a strong contribution to this axis, reflecting their discriminative role in capturing technical variability. In contrast, several compounds were strongly associated with PC2, including 2-methyl-5-(methylthio)-furan, 3-(hydroxymethyl)−2-nonanone, and 3-heptanone. These VOCs accounted for the clear separation between CM and TG samples from the culture medium, as well as for the partial yet consistent separation between CM and TG samples themselves, observed along PC2, which explained 17.06% of variance.

Focusing on the distribution of CM and TG samples along PC2 and PC3, explaining respectively 15.1% and 6.03% of the variance (Fig. 1C), and considering the corresponding loadings (Fig. 1D), several compounds were found to be positively correlated with PC3 and significantly different between the two classes. These included 2,6,11-trimethyl-dodecane, 3,3-dimethyl-hexane, dodecane, 3-methyl-octane, undecanone, and 2-ethylhexyl ester benzoic acid.

The impact of batch and treatment factors was further quantified using ASCA (Table 1). Before correction, the batch factor accounted for 58.88% of the variance and was highly statistically significant (*p*-value < 0.00001), whereas the treatment factor explained only 11.73% of the variance, although it was highly statistically significant (*p*-value = 0.0002). The interaction term contributed minimally (1.13%, *p*-value = 0.389). Overall, these results confirmed that batch was the dominant source of variability, partially obscuring treatment-related signals in the volatilome dataset.

**Table 1:** Results of ASCA model, including explained variance and statistical significance (*p*-values) of the experimental factors, TG treatment, batch, and their interaction

Factor	Pre-batch effect correction			Post-batch effect correction		
	no. PCs	Explained Variance [%]	*p*-value	no. PCs	Explained Variance [%]	*p*-value
Treatment	1	11.73	0.0002	1	47.12	< 0.00001
Batch	1	56.88	< 0.00001	1	0.16	1
Interaction	1	1.13	0.3892	1	7.73	0.0002

The application of EPO effectively removed the batch effect. After correction, the batch factor accounted for only 0.16% of the variance and was no longer statistically significant (*p*-value = 1), while the treatment factor became predominant, explaining 47.12% of the total variance with strong statistical significance (*p*-value < 0.00001). These findings indicate that the batch-related variability was successfully corrected. Notably, the interaction term also gained importance after correction, explaining 7.73% of the variance (*p*-value = 0.0002). This increase reflects the fact that, once the overwhelming batch signal was removed, variance could be redistributed more accurately across treatment-related components. In practical terms, this means that the volatilome response to TG includes both a robust direct effect and a modulated component that may vary across experimental replicates. Importantly, this does not indicate residual batch noise but rather a biological interaction**,** whereby the treatment effect is consistent yet partly influenced by the experimental context. In conclusion, the approach used in this study for batch effect correction proved to be effective and useful in the analysis of biological samples, particularly when it is important to include a large number of replicates while still accommodating the practical limitations of the experimental setup. Together, PCA and ASCA demonstrate that EPO effectively suppressed systematic batch-related noise, thereby enabling robust identification of biologically meaningful treatment effects.

### Exploratory analysis after batch effect correction

**Fig. 2:**
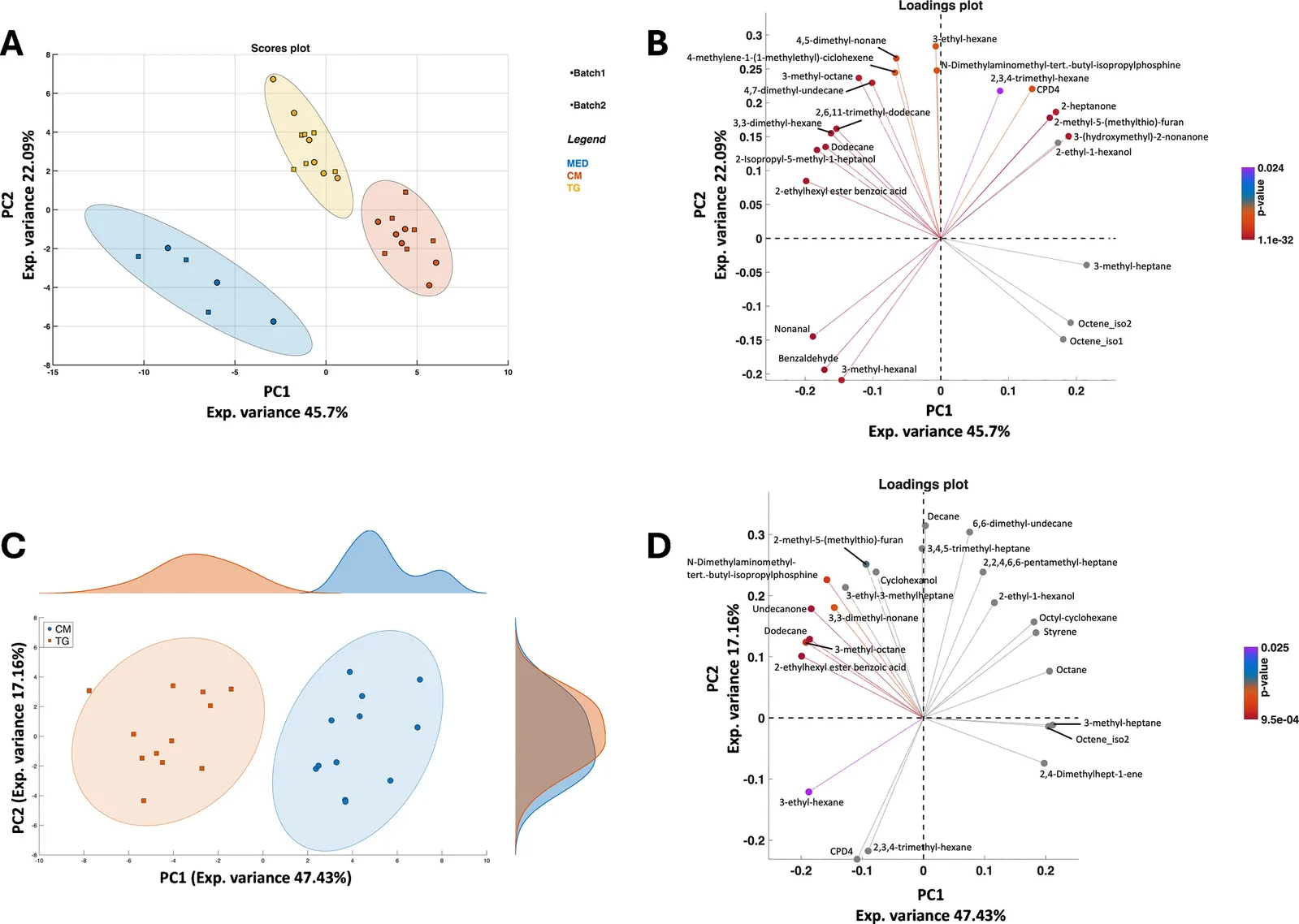
**A** PCA scores plot showing the distribution of data along PC1 and PC2, which explain respectively 45.7% and 22.09% of the total variance, after applying EPO correction. Score plot legend: culture medium (MED) blue, non-treated cells (CM) red, treated cells (TG) yellow. Batch 1 circles, Batch 2 squares. The confidence ellipses are based on Hotelling’s *T*^2^ statistic for a 95% confidence interval. They show the variability within each group and illustrate the distribution of data along the primary axes, indicating the direction of maximum variance. **B** PCA loadings plot highlighting the variables most responsible for group separation in the multivariate space along PC1 and PC2, displaying those with higher contribution to variance, coloured according to their *p*-values. **C** PCA scores plot showing the distribution of data along PC1 and PC2, which explain respectively 47.43% and 17.16% of the total variance, after EPO correction. Score plot legend: non-treated cells (CM) blue, treated cells (TG) red. The confidence ellipses are based on Hotelling’s *T*^2^ statistic for a 95% confidence interval. They show the variability within each group and illustrate the distribution of data along the primary axes, indicating the direction of maximum variance. **D** PCA loadings plot showing the contribution of each VOC to PC1 and PC2, displaying those with higher contribution to variance, coloured according to their *p*-values

After EPO correction, PCA (Fig. 2A) revealed a robust separation between CM and TG samples, as well as between the cell-derived samples and the culture media. In this corrected model, PC1 (45.7%) captured the treatment-driven variance, while PC2 (22.09%) accounted for residual variability. Five of the six VOCs mentioned above, i.e., 2,6,11-trimethyl-dodecane, 3,3-dimethyl-hexane, dodecane, 3-methyl-octane, and 2-ethylhexyl ester benzoic acid, were positively correlated with PC2 and therefore contributed to the separation between CM and TG samples along that axis, together with other compounds, such as 2-isopropyl-5-methyl-1-heptanol, and 4,7-dimethyl-undecane (Fig. 2B).

Among the compounds strongly associated with PC1, and thus accounting for the separation observed along the first PC among CM, TG, and culture medium samples, were 2-heptanone, 2-methyl-5-(methylthio)-furan, and 3-(hydroxymethyl)−2-nonanone, which were positively correlated with PC1. In contrast, nonanal, benzaldehyde, and 3-methyl-hexanal showed negative loadings along PC1 and were therefore more abundant in culture medium samples located at negative PC1 values.

When PCA was performed on CM and TG samples only (Fig. 2C), the separation between treated and untreated cells was further emphasised. In this analysis, dodecane, 3-methyl-octane, and 2-ethylhexyl ester benzoic acid remained positively correlated with PC2 and negatively correlated with PC1, confirming their role as key discriminants between the two classes (Fig. 2D). Additional statistically significant VOCs included undecanone, 3,3-dimethyl-nonane, and N-dimethylaminomethyl-ter-butyl-isopropylphosphine, suggesting that TG treatment induces a broad reprogramming of the volatilome beyond the most prominent contributors.

From a biochemical perspective, many of the discriminant compounds identified in this study, including alkanes and branched hydrocarbons (e.g., dodecane, 3-methyl-octane, 3,3-dimethyl-hexane) as well as aldehydes such as nonanal and benzaldehyde, are consistent with lipid peroxidation and altered aldehyde metabolism, processes known to be perturbed under endoplasmic reticulum stress [48]. The emergence of additional discriminant VOCs after correction suggests that TG treatment does not simply amplify pre-existing volatilome signatures but also induces novel metabolic outputs, potentially linked to broader cellular remodelling under stress conditions.

### Classification analysis

In order to characterise the differences between CM and TG cells, a PLS-DA classification model was developed and rigorously validated. The analysis was first performed on EPO-corrected data; however, since ASCA identified the treatment factor as statistically significant also for the raw (non-EPO-corrected) dataset, the same PLS-DA workflow was also applied to the raw data to compare the results and assess the robustness of the classification with respect to the pre-processing. Due to the relatively small dataset size, model validation was performed using a combination of rDCV and permutation testing. Given the structure of this rDCV procedure, the final model performance was expressed in terms of accuracy, sensitivity, and specificity, each averaged across 100 independent runs.

**Fig. 3:**
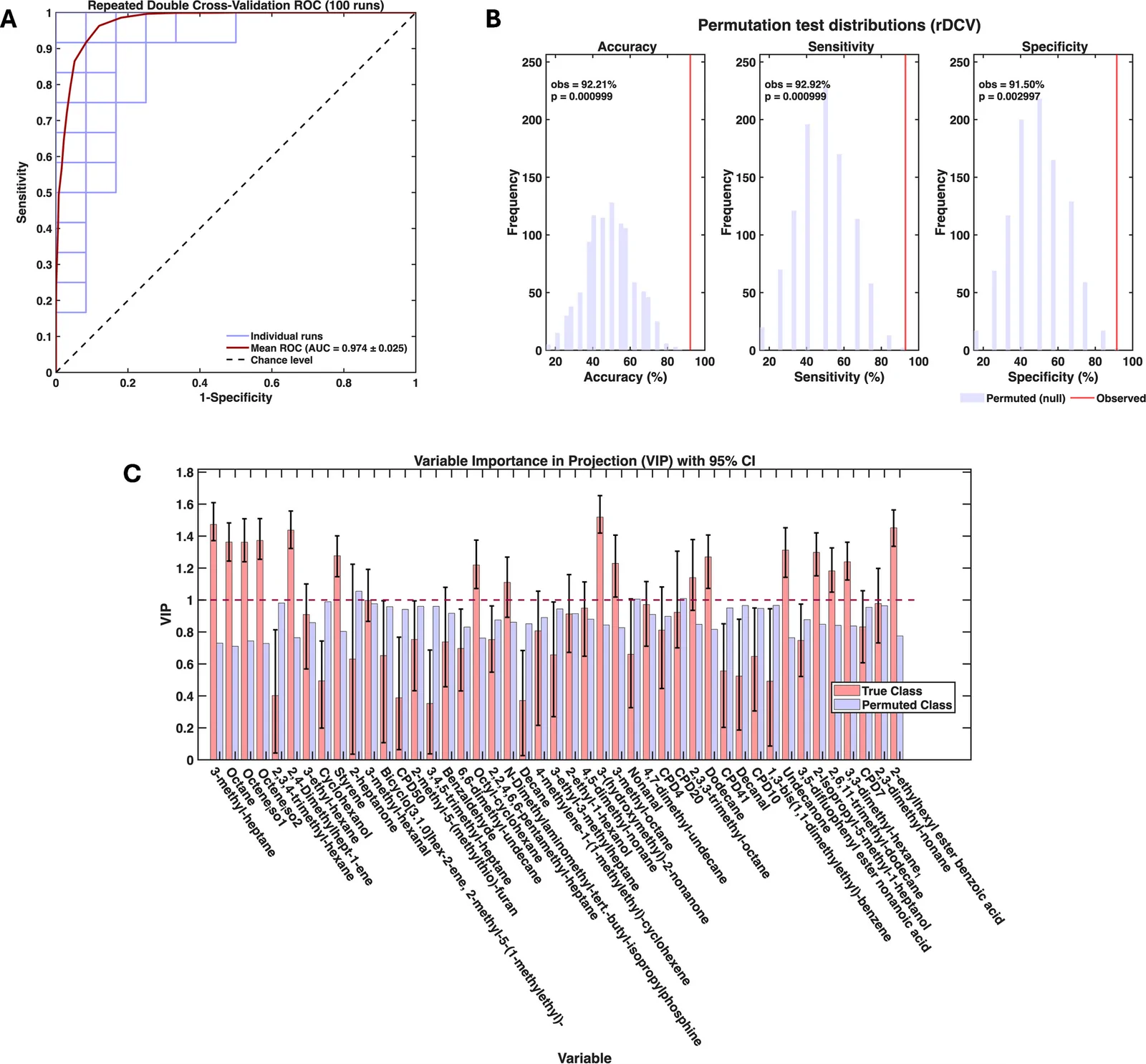
**A** Area under the curve (AUC) of the rDCV PLS-DA model with the EPO-corrected dataset, illustrating the classification performance. **B** Results of the permutation tests, showing the null distribution of accuracy (right), sensitivity (centre), and specificity (left) for the permuted models, compared with the performance of the true model (red line). In all three metrics, the original model lies outside the null distribution, indicating superior performance compared to permuted models. **C** VIP score plot for the original model (red bars) versus those from permuted models (lilac bars). For the 17 variables with VIP > 1, the VIP scores of the permuted models are consistently lower than those of the original model. The scores are reported as mean ± standard deviation

**Fig. 4:**
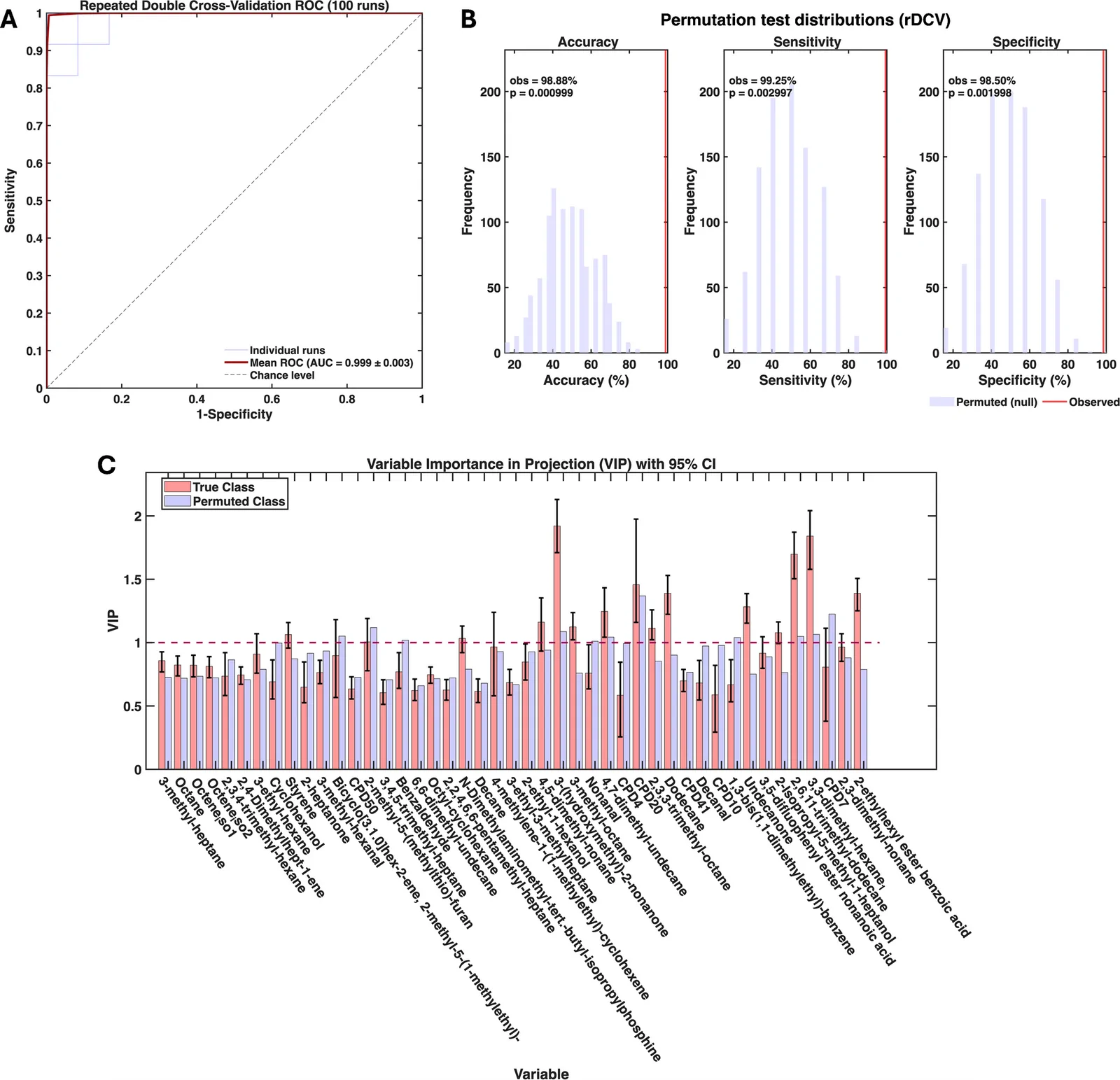
**A** Area under the curve (AUC) of the rDCV PLS-DA model with the raw dataset, illustrating the classification performance. **B** Results of the permutation tests, showing the null distribution of accuracy (right), sensitivity (centre), and specificity (left) for the permuted models, compared with the performance of the true model (red line). In all three metrics, the original model lies outside the null distribution, indicating superior performance compared to permuted models. **C** VIP score plot for the original model (red bars) versus those from permuted models (lilac bars). For the 15 variables with VIP > 1, the VIP scores of the permuted models are consistently lower than those of the original model. The scores are reported as mean ± standard deviation

The model achieved a mean overall accuracy of 92.21 ± 4.5%, which improved to 98.88 ± 0.025% for the raw dataset. The same trend was consistently observed across all the other performance metrics, including sensitivity, specificity, and AUC, with systematically higher values obtained for the raw dataset. Specifically, class-wise sensitivity (true positive rate) was 92.92 ± 5.35% (99.25 ± 0.024%, raw dataset) for the CM class, and 91.50 ± 6.27% (98.5 ± 0.038%, raw dataset) for the TG class, while specificity (true negative rate) was 91.50 ± 6.27% (98.5 ± 0.038%, raw dataset) for CM and 92.92 ± 5.35% (99.25 ± 0.024%, raw dataset) for TG. The mean AUC (Figs. 3A and 4A) across all runs was 0.974 ± 0.025 and 0.999 ± 0.002 for the raw dataset. These results indicate not only the robustness of the classification and the absence of overfitting but also suggest that batch correction may remove informative variance. Indeed, working on raw data outperformed batch-corrected data, likely because avoiding batch correction allows the model to capture natural sources of variability that are intrinsic to this type of experiment, where batch effects are often unavoidable due to the large number of samples and experimental sessions. Table 2 summarises PLS-DA model performance, and Figs. S3 and S4 show the cross-validated scores and loadings, with their 95% confidence intervals.

**Table 2:** Results of PLS-DA models, reported as overall accuracy, class-wise sensitivity and specificity, and ROC-AUC, for the EPO-corrected and raw datasets. Each metric was averaged across all 100 runs, and its confidence interval (SD %) is reported

	**EPO-corrected data**			**Raw data**		
	**Overall (SD) [%]**	**CM (SD) [%]**	**TG (SD)** **[%]**	**Overall (SD) [%]**	**CM (SD) [%]**	**TG (SD) [%]**
**Accuracy**	92.21 (4.50)	-	-	98.88 (0.025)	-	-
**Sensitivity**	-	92.92 (5.35)	91.50 (6.27)	-	99.25 (0.024)	98.5 (0.038)
**Specificity**	-	91.50 (6.27)	92.92 (5.35)	-	98.5 (0.038)	99.25 (0.024)
**AUC**	0.972 (0.025)	-	-	0.999 (0.003)	-	-

For both datasets, the robustness of the classification was further confirmed by permutation testing (1000 iterations), which demonstrated that the observed performance metrics (accuracy, sensitivity, specificity) consistently lay far outside the corresponding null distribution (Figs. 3B and 4B), with an empirical *p*-value of 0.001. These findings confirm that the predictive performance of the models is not attributable to random correlations but reflects genuine treatment-related metabolic differences.

To identify the metabolites most responsible for class discrimination, VIP scores with 95% confidence intervals were computed (Figs. 3C and 4C). For the corrected dataset, 17 variables exhibited VIP > 1 in at least 95% of the runs and were therefore retained for further consideration, ensuring that only consistently important features were included in the study. For the raw dataset, 15 VOCs showed a VIP > 1, 11 of which overlapped with those identified in the corrected dataset. Among these, 3-(hydroxymethyl)−2-nonanone exhibited the highest VIP score, followed by 2-ethylhexyl ester benzoic acid, dodecane, and undecanone. Additional common discriminant VOCs included 3-methyl-octane, 2,3,3-trimethyl-octane, 2-isopropyl-5-methyl-1-heptanol, styrene, 2,6,11-trimethyl-dodecane, N-dimethylaminomethyl-tert-butyl-isopropylphosphine, and 3,3-dimethyl-hexane, which also contributed substantially to group separation.

In both models, the rank product (RP) analysis (Figs. S5 and S6) and the RP ratio plot (Figs. S7 and S8) further confirmed the stability of the identified variables. Specifically, the RP consistently ranked the same set of VOCs among the top contributors in true-class models compared to permutations, while the RP ratio values were markedly below 1, highlighting their reproducibility and robustness across validation runs.

Taken together, the supervised PLS-DA results demonstrate that TG treatment induces a reproducible and highly discriminant volatilome signature in A375 cells. The combination of high predictive accuracy, stringent validation through permutation tests, and stable variable importance profiles provides strong evidence that the identified VOCs represent genuine treatment-related metabolic alterations.

The two PLS-DA models were built to evaluate whether the observed separation between CM and TG cells depends on data pre-processing or reflects genuine biological differences. While EPO correction is useful to reduce technical variability, it can also modify data structure by removing informative variability. Analysing both datasets allowed us to assess the robustness of classification.

Both models showed excellent and statistically significant classification performance, as confirmed by rDCV and permutation testing. This indicates that the discrimination between CM and TG samples is strong and not attributable to random correlation. The better performance observed in the raw dataset suggests that class-related metabolic differences are already pronounced before correction, while the EPO-corrected model provides a more conservative approach that reduces potential technical biases.

The highly consistent results across the two models, with most of the discriminant VOCs in common, indicated that the key features driving class separation are largely independent of pre-processing.

Overall, the use of two parallel models strengthens confidence in both the classification performance and the identified discriminant VOCs, supporting their relevance for further biological interpretation and validation.

### Univariate analysis and volatile profile comparison

Once established that the two PLS-DA models exhibited comparable performance and identified largely overlapping sets of significant variables, univariate analysis was subsequently performed on the 17 key features selected by the EPO-corrected model (Table 3 and Fig. S9). The corresponding trend of the key VOCs in the raw dataset is reported in Fig. S10. Univariate comparison supported the biological interpretation of the results by enabling the identification of compounds that are either produced or consumed by the cellular metabolism in both treated and untreated cells.

**Table 3:** List of VOCs and their relative abundance in each class, expressed with respect to the medium (M): ↑ increased, ↓ decreased,—no significant difference. The last three columns indicate the statistical significance of pairwise comparisons between groups. *p < 0.05, **p < 0.001. *ns*, non-significant. Compounds marked as “unknown” were not identified with a match score ≥ 80%

	**Trend**			**Significance**		
**Compound**	**M**	**CM**	**TG**	**M vs CM**	**M vs TG**	**CM vs TG**
3-Methyl-heptane	↓	↑	↑	**	*	**
Octane	↓	↑	-	**	n.s	**
2-Octene (isomer 1)	↓	↑	-	**	n.s	**
2-Octene (isomer 2)	↓	↑	-	**	n.s	**
2,4-Dimethylhept-1-ene	↓	↑	↑	**	**	**
Styrene	↓	↑	↑	**	**	**
Octyl-cyclohexane	↓	↑	↑	**	*	*
N-Dimethylaminomethyl-ter-butyl-isopropylphospine	↓	**-**	↑	n.s	**	**
3-(Hydroxymethyl)−2-nonanone	↓	↑	↑	**	**	**
3-Methyl-octane	↑	↓	-	*	n.s	**
2,3,3-Trimethyl-octane	↑	↓	-	**	n.s	**
Dodecane	↑	↓	-	*	n.s	**
Undecanone	↑	↓	↓	**	*	**
2-Isopropyl-5-methyl-1-heptanol	↑	↓	-	**	n.s	**
2,6,11-Trimethyl-dodecane	↑	↓	-	**	n.s	**
3,3-Dimethyl-hexane	↑	↓	-	**	n.s	**
2-Ethylhexyl ester benzoic acid	↑	↓	↓	**	*	**

Specifically, compounds exhibiting higher concentrations in both CM and TG compared to the culture medium are likely metabolic products common to both treated and untreated melanoma cells. Conversely, VOCs detected at lower concentrations than in the medium are likely consumed by cellular metabolism. Moreover, any increase or decrease in their abundance in one or both cell classes may reflect the upregulation or downregulation of specific metabolic pathways in those cells.

Cancer cells are known to exhibit higher basal levels of oxidative stress compared to healthy cells, as a consequence of altered metabolism and increased ROS production. These ROS promote peroxidation of polyunsaturated fatty acids (PUFA), leading to the formation of methylated hydrocarbons, which can subsequently be oxidised by cytochrome P450 enzymes into alcohols, ketones, and aldehydes [20, 21, 49–51].

In this context, 2-ethylhexyl ester benzoic acid was detected at higher concentrations in the culture medium than in either cell type. Among CM and TG, this compound was more abundant in TG-treated cells, suggesting increased consumption in untreated cells. As this ester can undergo enzymatic hydrolysis by carboxylesterase, producing benzoic acid and 2-ethyl-1-hexanol [52, 53], these findings may indicate enhanced activity of this enzyme family in untreated cells. This interpretation is further supported by the higher concentration of 2-ethyl-1-hexanol, one of the products of this reaction, found in CM cells compared to both TG and the culture medium (Fig. S11), although this compound did not rank among the most discriminant variables in the classification model.

Elevated levels of 2-ethyl-1-hexanol have previously been observed in the headspace of various lung cancer cell lines compared with either culture medium or healthy cell cultures [54, 55]. Furthermore, Fenn et al. (2023) reported an increased release of 2-ethyl-1-hexanol concentration in the headspace of A549 lung cancer cells exposed to hydrogen peroxide to induce oxidative stress, compared to the blank culture medium [51]. Taken together, these observations may suggest that the higher level of 2-ethyl-1-hexanol in untreated cells may be driven not only by enhanced carboxylesterase activity but also by increased oxidative stress and lipid peroxidation.

A similar trend to that observed for 2-ethylhexyl ester benzoic acid was found for undecanone, which displayed significant differences among the culture medium, CM, and TG. This ketone has been previously detected at elevated levels in two human gastric cancer cell lines, but it was absent in normal gastric mucosa cells [56].

Six VOCs were detected at higher concentrations in the culture medium compared to CM cells, but not compared to TG, while significant differences were observed between CM and TG. This pattern indicates that these compounds cannot be directly attributed to production by treated cells but reflect metabolic differences between treated and untreated cells, potentially due to different uptake, transformation, or release of these compounds. Most of these VOCs are linear or branched alkanes, including 3-methyl-octane, 2,3,3-trimethyl-octane, dodecane, 2,6,11-trimethyl-dodecane, and 3,3-dimethyl-hexane, which are frequently reported in VOC studies as markers of background contamination or as compounds influenced by medium-cell interactions rather than direct cellular biosynthesis [57, 58]. For example, dodecane has been reported in the headspace of colorectal cancer tumour tissues without a significant difference relative to normal tissue [59]; the same compound was also found in significantly high concentrations in the headspace of human mantle cell lymphoma cells, but it was also detected in the headspace of human lymphocyte cells and of culture medium blank [60]. Similarly, the presence of 2-isopropyl-5-methyl-1-heptanol, which is not commonly described as a direct product of cellular metabolism, is more likely attributable to differences in processing this compound between TG and CM cells rather than direct production.

In contrast, among compounds likely derived from lipid oxidation, 3-(hydroxymethyl)−2-nonanone and 3-methyl-heptane were detected at higher concentrations in CM cells compared to both TG and the culture medium, suggesting active production in untreated cells, potentially linked to increased oxidative stress. Although 2,4-dimethylhept-1-ene, styrene, and octyl-cyclohexane showed a similar trend, these compounds are not typically associated with endogenous cellular metabolism. Aromatic VOCs like styrene are indeed more likely to originate from environmental contamination, particularly from polymer- and copolymer-based materials (e.g., septa of headspace vial caps) [57, 61].

Lastly, octane, 2-octene (isomer 1), and 2-octene (isomer 2) were more abundant in CM than TG but did not differ significantly between TG and culture medium. To support this, decreased levels of 2-octene have previously been reported in H_2_O_2_-exposed human colon cancer cells and in the exposed culture media [9].

The identification of potentially exogenous compounds underscores the high sensitivity of the GC-MS platform and the importance of carefully distinguishing between endogenous metabolites and environmental contamination from manufacturing materials. Rather than representing a limitation, the proactive recognition of these artefacts represents a rigorous and transparent approach to data validation. By incorporating medium blanks and applying stringent comparative analyses, the present study accounts for background contributions and isolates a high-confidence metabolic signature. This strategy ensures that the reported differences between experimental groups reflect genuine cellular activity rather than analytical noise. Future studies would further benefit from comprehensive blanking strategies and standardised material selection, which not only mitigate background interference but also enhance the signal-to-noise ratio and improve the reproducibility and biological relevance of volatilome profiling.

## Conclusions

The HS-SPME/GC-MS approach employed in this study proved to be a robust and sensitive method for the characterisation of the volatilome of human melanoma cells. This analytical strategy enabled the detection of a broad spectrum of VOCs spanning multiple chemical classes, thereby providing valuable insight into cellular metabolic activity and stress-related responses. The non-invasive nature of HS analysis, combined with the selectivity of SPME and sensitivity of GC-MS, makes this approach particularly well suited for investigating subtle metabolic changes in cell-based models.

The integration of chemometric approaches played a crucial role in improving data quality and interpretability. In particular, the comparison of PLS-DA models built on batch-corrected and raw data indicated that batch correction effectively reduced variability associated with experimental batches without altering the underlying biological signal. This dual approach demonstrates that the observed discrimination is robust to pre-processing choices and confirms that the adopted multivariate workflow improves the reliability, robustness, and reproducibility of the identified biological trends.

Despite these methodological strengths, careful interpretation of volatilomic data remains essential. Not all detected VOCs can be attributed to endogenous cellular metabolism, as contributions from culture media, laboratory materials, and other environmental sources may be present. Misattributing such compounds to cellular activity could result in the overestimation of specific metabolic pathways or in misleading conclusions regarding treatment-induced effects.

For these reasons, meticulous blank analysis and rigorous comparative evaluation are essential to avoid misattributing external contaminations to cellular activity, a key challenge in volatilomics research.

## Supplementary Information

Below is the link to the electronic supplementary material.

Supplementary file1 (DOCX 4.89 MB)

## Data Availability

Data will be made available on request.
