# Supplementary material for: SPME-based investigation of thapsigargin-induced alterations in the volatilome of human melanoma cells
Source: Anal Bioanal Chem. 2026 Feb 4;418(18):5881–94. doi: 10.1007/s00216-026-06359-8 (PMC13541825; doi:10.1007/s00216-026-06359-8)
Supplement: Supplementary file 1 [file 216_2026_6359_MOESM1_ESM.docx]

**Supplementary Information (SI)**

**SPME-based Investigation of Thapsigargin-Induced Alterations in the Volatilome of Human Melanoma Cells**

*Elisabetta Santarelli^1,2 §^, Matteo Delli Carri^1,2 §^, Maria Rosaria Miranda^1,2^, Vicky Caponigro^1^, Vincenzo Vestuto^1^, Agnieszka Smolinska^3^, Andrea Manni^4^, Pietro Campiglia^1^, Giacomo Pepe^1^, Carlo Crescenzi^1*^*

^1^ Department of Pharmacy, University of Salerno, 84084 Fisciano, Salerno, Italy

^2^ PhD Program in Drug Discovery and Development, University of Salerno, 84084 Fisciano, Salerno, Italy

^3^ Department of Pharmacology and Toxicology, Maastricht University, Maastricht, The Netherlands

^4^ Spectra 2000 Srl, 00133 Rome, Italy

* Corresponding author

§ These authors contributed equally

**Fig. S1** Effects of thapsigargin (TG) on A375 cell viability. Viability of A375 cells, untreated or treated with the different concentrations of TG indicated on the X-axis. Cell viability after 24 h is reported. Data are expressed as mean ± standard deviation (SD) from n = 2 independent experiments. Statistical significance was determined by t-test (**p<0.01, ***p<0.001) for the indicated time points vs. the corresponding untreated control.

**Fig. S2** Effects of thapsigargin (TG) on A375 ER stress using the ThT (Thioflavin T) protein misfolding assay. ER stress was evaluated in A375 cells, untreated or treated with TG EC_50_ (~600 nM) at different time points indicated on the X-axis, recording the cell fluorescence. Data are expressed as mean ± standard deviation (SD) from n = 2 independent experiments. Statistical significance was determined by t-test (**p<0.01, ***p<0.001) for the indicated time points vs. the corresponding untreated control.

**Fig. S3** Results of the repeated double cross-validation procedure with the EPO-corrected dataset. **A)** Cross-validated scores with 95% confidence intervals (CI), showing separation between CM (red) and TG (blue) groups. **B)** Cross-validated weights with 95% CI, indicating the contribution and stability of individual variables to the discriminant function.

**Fig. S4** Results of the repeated double cross-validation procedure with the raw dataset. **A)** Cross-validated scores with 95% confidence intervals (CI), showing separation between CM (red) and TG (blue) groups. **B)** Cross-validated weights with 95% CI, indicating the contribution and stability of individual variables to the discriminant function.

**Fig. S5** Rank product (RP) comparison between true class (red) and permuted class (blue) for the analysed variables in the EPO-corrected dataset. Higher RP values in the true class indicate compounds with stronger discriminatory power compared to the random (permuted) distribution, supporting their relevance in class separation*.*

**Fig. S6** Rank product (RP) comparison between true class (red) and permuted class (blue) for the analysed variables in the raw dataset. Higher RP values in the true class indicate compounds with stronger discriminatory power compared to the random (permuted) distribution, supporting their relevance in class separation*.*

**Fig. S7** Rank product (RP) ratio plot showing the stability of variable ranking in the EPO-corrected dataset. Higher RP ratio values indicate variables with consistent and robust discriminative power across repeated resampling, highlighting their reliability for class separation.

**Fig. S8** Rank product (RP) ratio plot showing the stability of variable ranking in the raw dataset. Higher RP ratio values indicate variables with consistent and robust discriminative power across repeated resampling, highlighting their reliability for class separation.

**Fig. S9** Boxplots showing the distribution in TG-treated cells (TG), untreated cells (CM), and cell-free medium (M) of the significantly relevant VOCs, according to the VIP scores of PLS-DA models for the EPO-corrected dataset. *p-value<0.05 and **p-value<0.001. **A)** 3-methyl-heptane; **B)** Octane; **C)** Octene (isomer 1); **D)** Octene (isomer 2); **E)** 2,4-dimethylhept-1-ene; **F)** Styrene; **G)** Octyl-cyclohexane; **H)** N-dimethylaminomethyl-ter-butyl-isopropylphospine; **I)** 3-(hydroxymethyl)-2-nonanone; **J)** 3-methyl-octane; **K)** 2,3,3-trimethyl-octane; **L)** Dodecane; **M)** Undecanone; **N)** 2-isopropyl-5-mehtyl-1-heptanol; **O)** 2,6,11-trimethyl-dodecane; **P)** 3,3-dimethyl-hexane; **Q)** 2-ethylhexyl ester benzoic acid.

**Fig. S10** Boxplots showing the distribution in TG-treated cells (TG), untreated cells (CM), and cell-free medium (M) of the significantly relevant VOCs, according to the VIP scores of PLS-DA models for the raw dataset. *p-value<0.05 and **p-value<0.001. **A)** Styrene; **B)** N-dimethylaminomethyl-ter-butyl-isopropylphospine; **C)**3-(hydroxymethyl)-2-nonanone; **D)** 3-methyl-octane; **E)** 2,3,3-trimethyl-octane; **F)** Dodecane; **G)** Undecanone; **H)** 2-isopropyl-5-mehtyl-1-heptanol; **I)** 2,6,11-trimethyl-dodecane; **J)** 3,3-dimethyl-hexane; **K)** 2-ethylhexyl ester benzoic acid; **L)** 2-methyl-5-(methylthio)-furan; **M)** 4,7-dimethyl-undecane; **N)** 4,5-dimethyl-nonane; **O)** CPD20 (unknown).

**Fig. S11** Boxplots showing the distribution in TG-treated cells (TG), untreated cells (CM), and cell-free medium (M) of 2-ethyl-1-hexanol, for the EPO-corrected dataset. *p-value<0.05 and **p-value<0.001.
